# Prediction of decreased estimated glomerular filtration rate using liver fibrosis markers: a renal biopsy-based study

**DOI:** 10.1038/s41598-022-22636-9

**Published:** 2022-10-21

**Authors:** Akira Mima

**Affiliations:** Department of Nephrology, Osaka Medical and Pharmaceutical University, Osaka, 569-8686 Japan

**Keywords:** Nephrology, Kidney, Kidney diseases

## Abstract

Non-alcoholic fatty liver disease is the most common chronic liver disease and is associated with chronic kidney disease. The fibrosis-4 index and non-alcoholic fatty liver disease score are widely used as non-invasive diagnostic methods for non-alcoholic fatty liver disease. However, the relationship between these markers and specific renal histopathologies in chronic kidney disease remain unclear. This study included 179 patients aged between 16 and 80 years who underwent renal biopsy. We examined the association between the fibrosis-4 index or non-alcoholic fatty liver disease score and change in estimated glomerular filtration rate 12 months after kidney biopsy for each renal histopathology. Renal histopathologies were determined by renal biopsy. Our results showed that there was a significant negative correlation between the fibrosis-4 index and estimated glomerular filtration rate. In nephrosclerosis, the non-alcoholic fatty liver disease score and estimated glomerular filtration rate tended to have a negative correlation, albeit without significance. In IgA nephropathy, both the fibrosis-4 index and non-alcoholic fatty liver disease score were significantly negatively correlated with estimated glomerular filtration rate. Furthermore, the fibrosis-4 index was not associated with urinary protein-to-creatinine ratio or renal function markers such as urinary b2 microglobulin and urinary *N*-acetyl-d-glucosamine. Our kidney biopsy-based study showed that the liver fibrosis markers fibrosis-4 index and non-alcoholic fatty liver disease score were negatively correlated with the estimated glomerular filtration rate in nephrosclerosis and IgA nephropathy.

## Introduction

Chronic kidney disease is associated with increased risks of mortality and morbidity. Furthermore, CKD is also associated with diabetes mellitus, including diabetic kidney disease (DKD)^1^. Obesity and insulin resistance are the most common causes of CKD^2,3^. It has been reported that non-alcoholic fatty liver disease (NAFLD) is a clinicopathological condition that encompasses a variety of chronic liver diseases ranging from asymptomatic hepatic fat accumulation to progressive liver disease, with clinical symptoms resembling alcoholic liver disease despite the absence of excessive alcohol consumption^4,5^. A recent study revealed that the prevalence of NAFLD diagnosed by ultrasound was estimated to be about 25%^6^. It is estimated that either NAFLD or non-alcoholic steatohepatitis (NASH) will be the most common indication for liver transplantation by 2030^7^. NAFLD is a multisystem disease that is associated with various diseases such as diabetes mellitus, obesity, metabolic syndrome, cardiovascular diseases, and CKD^8^. The fibrosis-4 (FIB-4) index and NAFLD fibrosis score (NFS) are simple and validated diagnostic markers used to stratify the degree of liver fibrosis according to risk in patients with suspected NAFLD^9^. Moreover, these non-invasive fibrosis markers have been shown to be associated with the prevalence of CKD in recent studies^10^. However, there have been no reports on the association between these fibrosis markers and decreased estimated glomerular filtration rate (eGFR) by renal histopathology classification as determined by kidney biopsy. Thus, we performed this renal pathology-based study to elucidate whether the FIB-4 index and NFS can predict eGFR decline in CKD patients.

## Results

We determined the clinical characteristics of the 179 cases after excluding patients who underwent kidney biopsy. The median age of the study participants was 51 years, and 96 patients (53.6%) were female. The median body mass index was 22.7, and the median FIB-4 index was 0.96. The other median values are as follows: systolic blood pressure (SBP), 129 mmHg; diastolic blood pressure (DBP), 78 mmHg; serum creatinine (Cr), 76.02 mmol/L; blood urea nitrogen (BUN), 5 mmol/L; estimated glomerular filtration rate (eGFR), 64 mL/min/1.73 m^2^; urine protein-to-creatinine ratio, 0.538; *N*-acetyl-β-d-glucosaminidase (urinary NAG), 8.9 IU/L; and urinary β2 microglobulin (urinary β2 MG), 198 mg/L (Table 1). The underlying diseases are as follows: nephrosclerosis (10 cases, 5.6%), IgA nephropathy (74 cases, 41.3%), minimal change disease (24 cases, 13.4%), diabetic nephropathy (7 cases, 3.9%), lupus nephritis (12 cases, 6.7%), membranous nephropathy (18 cases, 10.1%), ANCA-associated glomerulonephritis (9 cases, 5.0%), interstitial nephritis (9 cases, 5.0%), and others (16 cases, 8.9%) (Table 2).

**Table 1:** Patient characteristics.

Variables	All patients (n = 179)
Age (years)	51 (16–80)
Female gender	96 (53.6%)
Body mass index	22.7 (14.3–45.7)
SBP (mmHg)	129 (90–236)
DBP (mmHg)	78 (40–142)
BUN (mmol/L)	5 (2.5–35.7)
Serum creatinine (µmol/L)	76.02 (26.69–38.13)
eGFR (mL/min/1.73m^2^)	64 (9–140)
Urinary β2 MG (mg/L)	198 (47–73,162)
Urinary NAG (IU/L)	8.9 (1.0–221.1)
Urine protein/creatine ratio	0.538 (0.04–28.86)
FIB-4 index	0.96 (0.19–6.58)
NFS	− 1.78 (− 4.88 to − 2.74)

*SBP* systolic blood pressure, *DBP* diastolic blood pressure, *BUN* blood urea nitrogen, *eGFR* estimated glomerular filtration rate, *urinary β2 MG* urinary β2 microglobulin, *urinary NAG* urinary *N*-acetyl-β-d-glucosaminidase, *FIB-4 index* fibrosis-4 index, *NFS* non-alcoholic fatty liver disease score.

**Table 2:** Underlying diseases in study.

Underlying disease	Number (%)
Nephrosclerosis	10 (5.6)
IgA nephropathy	74 (41.3)
Minimal change disease	24 (13.4)
Diabetic nephropathy	7 (3.9)
Lupus nephritis	12 (6.7)
Membranous nephropathy	18 (10.1)
ANCA-associated glomerulonephritis	9 (5.0)
Interstitial nephritis	9 (5.0)
Others	16 (8.9)

**Figure 1:**
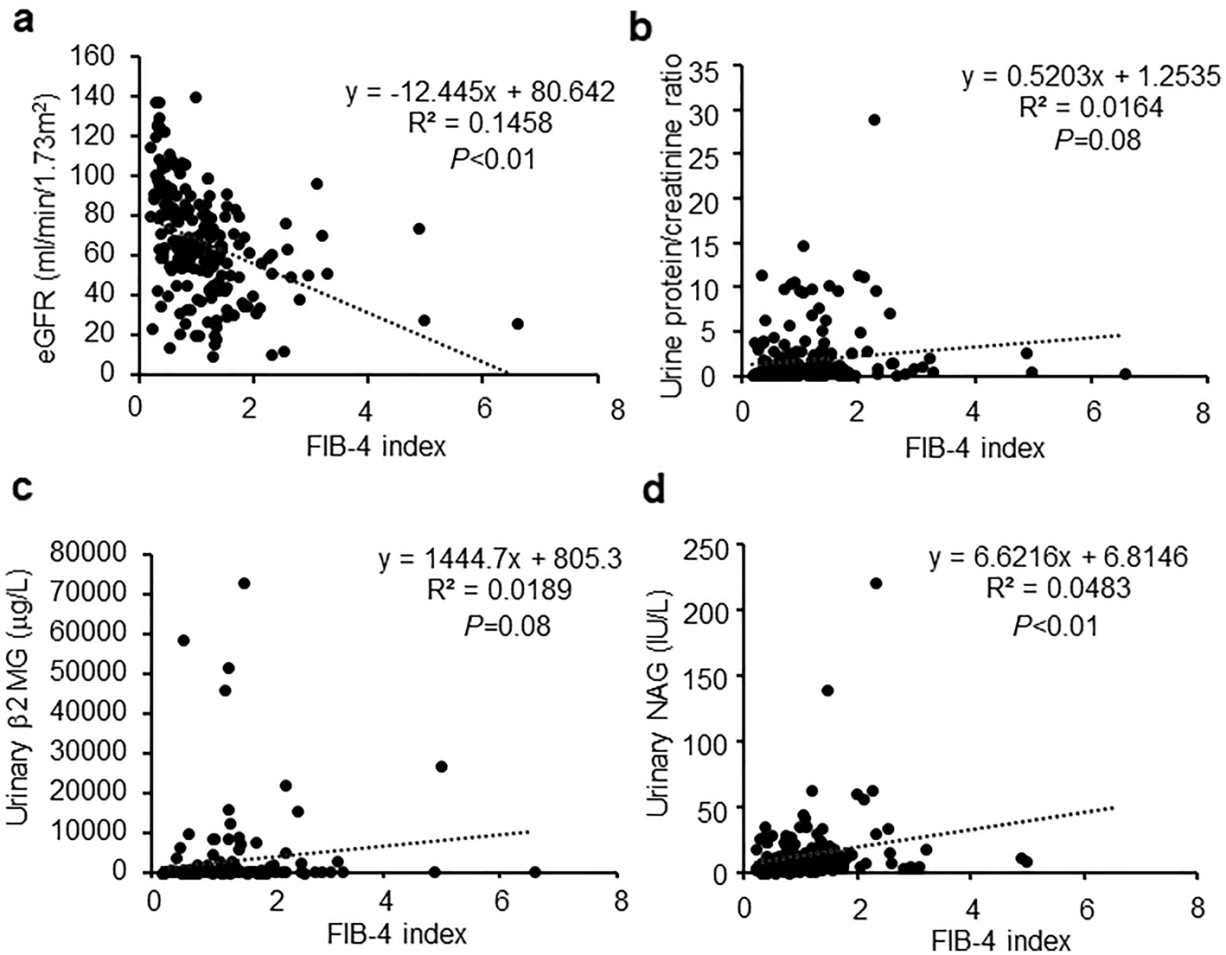
Associations of the FIB-4 index and renal function markers. (**a**) Correlation between the FIB-4 index and estimated glomerular filtration rate. (**b**) Correlation between the FIB-4 index and urine protein-creatinine ratio. (**c**) Correlation between the FIB-4 index and urinary b2 microglobulin. (**d**) Correlation between the FIB-4 index and urinary *N*-acetyl-b-d-glucosaminidase.

We calculated the FIB-4 index and eGFR before renal biopsy for all diseases. The results showed that there was a weak but negative correlation between the FIB-4 index and eGFR (R^2^ = 0.1458, *P* < 0.01, Fig. 1a). In contrast, the FIB-4 index was not correlated with the urinary protein-to-creatinine ratio (R^2^ = 0.0164, *P* = 0.08, Fig. 1b) and urinary β2 MG (R^2^ = 0.0819, *P* = 0.08, Fig. 1c). Furthermore, there was a negative correlation between the FIB-4 index and urinary NAG (R^2^ = 0.0483, *P* < 0.01, Fig. 1d).

**Figure 2:**
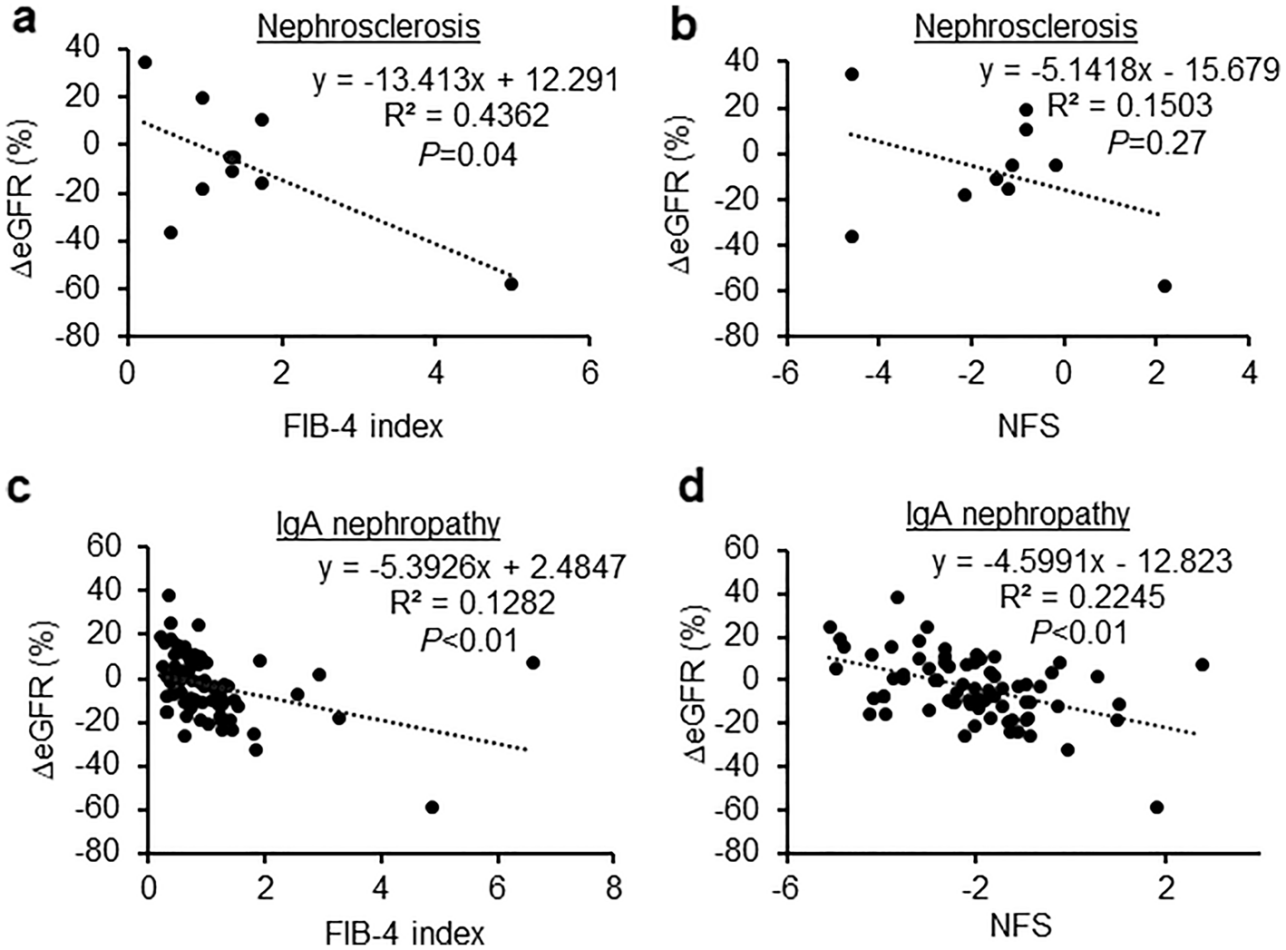
Associations between the change in estimated glomerular filtration rate and fibrosis markers in nephrosclerosis and IgA nephropathy. (**a**,**b**) Correlation between the change in the estimated glomerular filtration rate and the FIB-4 index (**a**) and NFS (**b**) in nephrosclerosis. (**c**,**d**) Correlation between the change in the estimated glomerular filtration rate and the FIB-4 index (**c**) and NFS (**d**) in IgA nephropathy.

**Figure 3:**
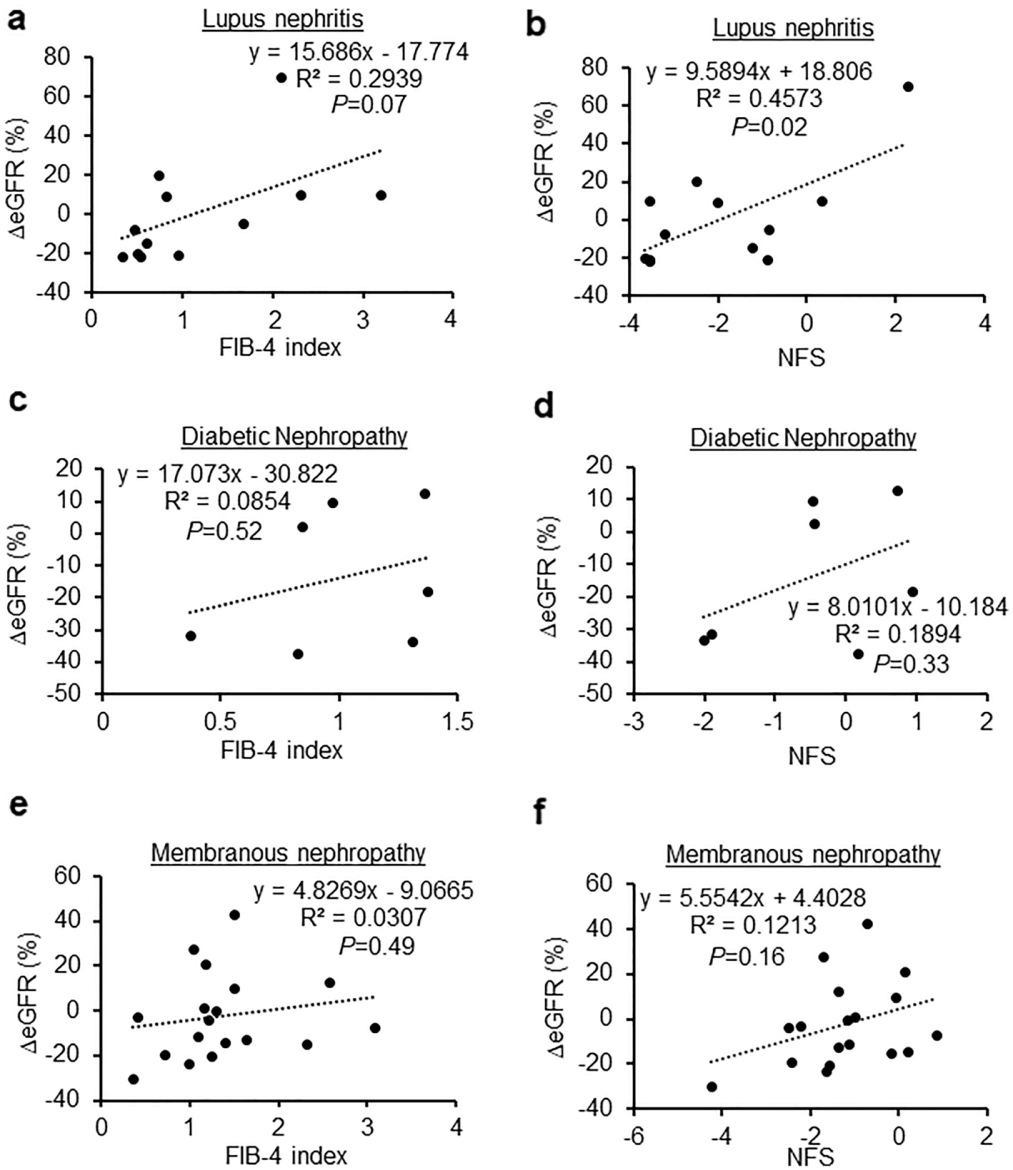
Associations between the change in estimated glomerular filtration rate and fibrosis markers in lupus nephritis, diabetic nephropathy, and membranous nephropathy. (**a**,**b**) Correlation between the change in the estimated glomerular filtration rate and the FIB-4 index (**a**) and NFS (**b**) in lupus nephritis. (**c**,**d**) Correlation between the change in the estimated glomerular filtration rate and the FIB-4 index (**c**) and NFS (**d**) in diabetic nephropathy. (**e**,**f**) Correlation between the change in the estimated glomerular filtration rate and the FIB-4 index (**e**) and NFS (**f**) in membranous nephropathy.

**Figure 4:**
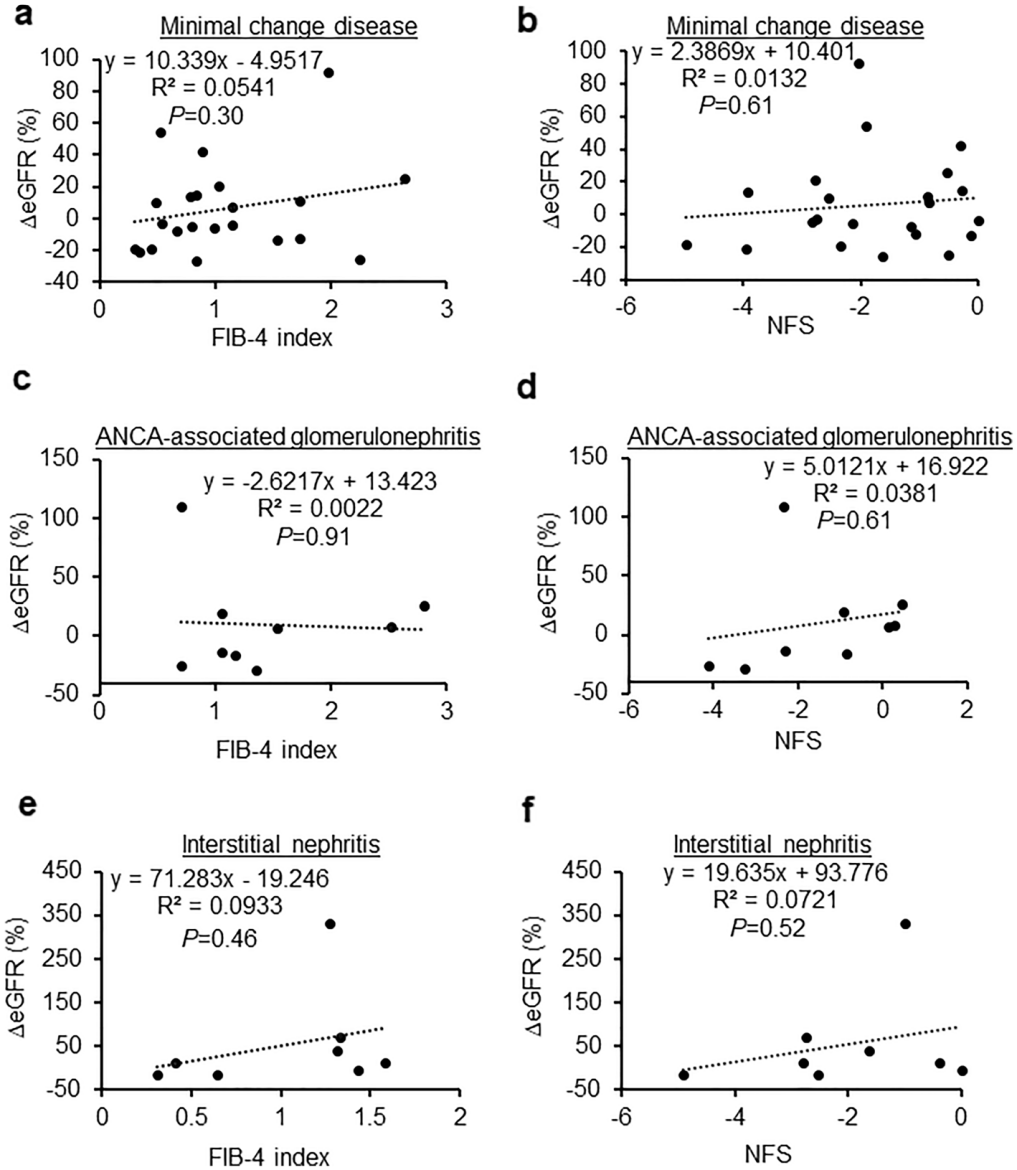
Associations between the change in estimated glomerular filtration rate and fibrosis markers in minimal change disease, ANCA-associated glomerulonephritis, and interstitial nephritis. (**a**,**b**) Correlation between the change in the estimated glomerular filtration rate and the FIB-4 index (**a**) and NFS (**b**) in minimal change disease. (**c**,**d**) Correlation between the change in the estimated glomerular filtration rate and the FIB-4 index (**c**) and NFS (**d**) in ANCA-associated glomerulonephritis. (**e**,**f**) Correlation between the change in the estimated glomerular filtration rate and the FIB-4 index (**e**) and NFS (**f**) in interstitial nephritis.

Next, we examined the relationship between FIB-4 index and NFS, and the rate of change in eGFR before and 12 months after renal biopsy. In nephrosclerosis, we found that the FIB-4 index was significantly negatively correlated with the eGFR (R^2^ = 0.4362, *P* = 0.04, Fig. 2a). Additionally, the correlation between NFS and eGFR tended to be negative, albeit without statistical significance (R^2^ = 0.1503, *P* = 0.27, Fig. 2b). In IgA nephropathy, the FIB-4 index (R^2^ = 0.1282, *P* < 0.01, Fig. 2c) and NFS (R^2^ = 0.2245, *P* < 0.01, Fig. 2d) were significantly negatively correlated with the eGFR. The rate of change in eGFR was not negatively associated with either FIB-4 or NFS in lupus nephritis (R^2^ = 0.2939, *P* = 0.07; R^2^ = 0.4573, *P* = 0.02, respectively; Fig. 3a,b), diabetic nephropathy (R^2^ = 0.0854, *P* = 0.52; R^2^ = 0.1894, *P* = 0.33, respectively; Fig. 3c,d), membranous nephropathy (R^2^ = 0.0307, *P* = 0.49; R^2^ = 0.1213, *P* = 0.16, respectively; Fig. 3e,f), minimal change disease (R^2^ = 0.0541, *P* = 0.30; R^2^ = 0.0132, *P* = 0.61, respectively; Fig. 4a,b), ANCA-associated glomerulonephritis (R^2^ = 0.0022, *P* = 0.91; R^2^ = 0.0381, *P* = 0.61, respectively; Fig. 4c,d), or interstitial nephritis (R^2^ = 0.0933, *P* = 0.46; R^2^ = 0.721, *P* = 0.52, respectively; Fig. 4e,f).

**Figure 5:**
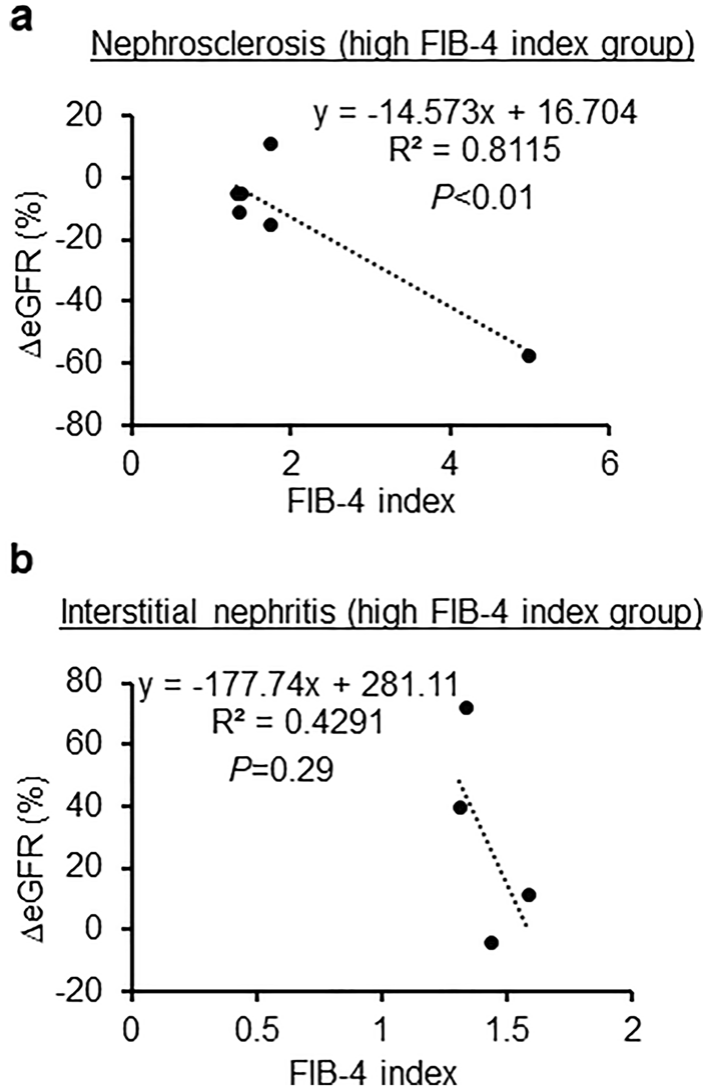
Associations between the change in estimated glomerular filtration rate and FIB-4 index in nephrosclerosis and IgA nephropathy of high FIB-4 index group. (**a**) Nephrosclerosis in high FIB-4 index group. (**b**) IgA nephropathy in high FIB-4 index group.

Next, we have checked in the high FIB-4 index group (≥ 1.3). In nephrosclerosis, we found that the FIB-4 index was more significantly negatively correlated with the rate of change in eGFR when examined in all cases including the low FIB-4 index (< 1.3) (R^2^ = 0.8115, *P* < 0.01, Fig. 5a). A similar trend was observed in interstitial nephritis, but the difference was not statistically significant (R^2^ = 0.4291, *P* = 0.29, Fig. 5b). However, other diseases did not show any changes when classified by high FIB-4 index levels.

The association between the rate of change in eGFR and the levels of Cr, BUN, and urinary β2 MG before kidney biopsy was revealed by multivariate analysis (Table 3). As shown in Table 4, multivariate analysis showed that the rate of change in eGFR and FIB-4 index were independently associated in both nephrosclerosis and IgA nephropathy, even adjusting for relevant factors.

**Table 3:** Multivariate analysis of factors associated with the rate of change in estimated glomerular filtration rate.

Variables	β value	SE value	*F* value	95% CI	*P* value
Age (years)	− 0.027	0.039	0.481	− 0.105–0.050	0.49
Body mass index	0.002	0.007	0.057	− 0.013–0.016	0.81
SBP (mmHg)	− 0.036	0.051	0.505	− 0.137–0.064	0.48
DBP (mmHg)	− 0.051	0.033	2.387	− 0.116–0.014	0.12
Serum creatinine (µmol/L)	0.006	0.001	19.023	0.003–0.009	< 0.01
BUN (mmol/L)	0.093	0.023	17.142	0.049–0.138	< 0.01
Urine protein/creatine ratio	0.001	0.001	0.011	− 0.014–0.016	0.92
Urinary NAG (IU/L)	0.067	0.051	1.707	− 0.034–0.169	0.19
Urinary b2MG (µg/L)	71.799	19.631	13.376	33.041–110.556	< 0.01

*SE* standard error, *CI* confidential interval, *SBP* systolic blood pressure, *DBP* diastolic blood pressure, *BUN* blood urea nitrogen, *urinary* β*2 MG* urinary β2 microglobulin, *urinary NAG* urinary *N*-acetyl-β-d-glucosaminidase.

**Table 4:** Multivariate analysis between the rate of change in estimated glomerular filtration rate and FIB-4 index in each renal pathology.

Variables	β value	SE value	*F* value	95% CI	*P* value
Nephrosclerosis	− 13.413	5.391	6.190	− 25.846 to − 0.981	0.04
IgA nephropathy	− 5.393	1.657	10.588	− 8.696 to − 2.088	< 0.01
Lupus nephritis	0.019	0.009	4.163	− 0.002–0.039	0.07
Diabetic nephropathy	0.005	0.007	0.467	− 0.014–0.024	0.52
Membranous nephropathy	4.827	6.782	0.506	− 9.551–19.205	0.49
Minimal change disease	10.339	9.670	1.143	− 9.832–30.511	0.30
ANCA-associated glomerulonephritis	− 2.621	21.291	0.015	− 52.969–47.726	0.91
Interstitial nephritis	71.283	90.700	0.618	− 150.651–293.217	0.46

*SE* standard error, *CI* confidential interval.

## Discussion

In the present study, we examined the relationship between each renal histopathology as determined by biopsy and non-invasive fibrosis markers. In nephrosclerosis, we found a statistically significant negative correlation between the FIB-4 index and eGFR, whereas the NFS and eGFR tended to have a negative correlation, albeit insignificant. Furthermore, in IgA nephropathy, both the NFS and FIB-4 index had statistically significant negative correlations with eGFR. Meanwhile, the pre-renal biopsy eGFR and FIB-4 index were negatively correlated in all subjects. This is consistent with previous reports, since patients eligible for renal biopsy suffered from CKD.

Our results showed that there was an association, albeit very weak, between the FIB-4 index and changes in eGFR. This may be due to the wide range of eGFR (1–100 ml/min/1.73 m^2^ or even higher), resulting in greater variability.

Recent studies have indicated that NAFLD could affect CKD, including DKD^11^. The FIB-4 index and NFS are widely used hepatic fibrosis markers for the diagnosis of NAFLD or NASH. Furthermore, the FIB-4 index could be useful for the prediction of the development of cardiovascular diseases^12^. It has been reported that the FIB-4 index is an indicator of inflammation; FIB-4 index is calculated using the platelet count, which contributes to thrombotic inflammatory actions owing to their ability to functionally interact with activated endothelial cells, leukocytes, and coagulation-related proteins^13^. Several studies have shown that inflammation is involved in the development of CKD^14,15^. As previously reported, nuclear factor NF-kappa-B (NF-kB) acts via inflammation and protein kinase C to activate the expression of cytokines; NF-kB is transferred to the nucleus, promptly activating the subsequent transcription of tumor necrosis factor-α, vascular cell adhesion molecule 1, or interleukin-6 in the kidneys of CKD patients^3,16,17^.

Previous reports have indicated that the activation of the TGF-β/Smad pathway is associated with the FIB-4 index and NFS^18^. We reported that Smad1 signaling plays a significant role in increasing the extracellular matrix, such as type 4 collagen and α-smooth muscle actin, in mesangial cells^19^. Inflammatory processes inevitably cause damage; as part of the healing process, glomerulosclerosis increases the mesangial extracellular matrix, leading to a decreased glomerular filtration rate^14,20^. Our results showed that the FIB-4 index and NFS were negatively correlated with eGFR in nephrosclerosis and IgA nephropathy, which are both characterized by an increase in mesangial extracellular matrix.

Obesity is the most common risk factor for NAFLD and an independent risk for developing CKD^21^. Furthermore, we have reported the mechanisms of developing obesity-related CKD^3,14–16,22^. NAFLD is one of the most common causes of chronic liver disease in clinical practice in its entire spectrum of conditions, ranging from simple lipidosis to steatohepatitis and cirrhosis^23–25^. It has been reported that liver fibrosis and fatty liver are crucial in the development of DKD^26^. In contrast, another study using multivariate analysis showed that the presence of NAFLD was not a significant predictor of the development of DKD^11^. In this regard, the association between NAFLD and DKD remains inconsistent. Although patients in our study tended to be obese (median value of body mass index: 22.7), we could not find any correlation between the FIB-4 index and eGFR in patients with type 2 diabetes.

Unlike eGFR, there was no clear correlation between urinary protein or tubulointerstitial markers and FIB-4 index. It has been reported that the FIB-4 index may be associated with vascular endothelial function^27^, which is a mechanism of glomerulosclerosis caused by glomerular endothelial dysfunction^3,17^. Thus, the FIB-4 index may be more reflective of glomerulosclerotic lesions. In contrast, proteinuria, which is mainly caused by podocyte dysfunction, may not be associated with the FIB-4 index, which is associated with glomerular endothelial cells^28,29^.

This study has several limitations. First, liver diseases, such as NASH or NAFLD, were not evaluated by imaging and liver biopsy. Second, we conducted a retrospective analysis of a cohort from a single center. Finally, some diseases, especially nephrosclerosis, had a small number of cases.

## Conclusion

The FIB-4 index and NFS, which are non-invasive fibrosis markers, could be predictive markers of reduced eGFR, especially in nephrosclerosis and IgA nephropathy.

## Material and methods

### Patient groups

All procedures performed in studies involving human participants were in accordance with the ethical standards of the institutional and/or national research committee and with the 1964 Helsinki Declaration and its later amendments or comparable ethical standards. The ethics committee of Osaka Medical and Pharmaceutical University approved this study (approval number: 2020-095). Written informed consent was obtained from all subjects. This study included a retrospective cohort of 179 cases who underwent kidney biopsy at Osaka Medical and Pharmaceutical University Hospital between April 2015 and July 2020. Data were collected and analyzed retrospectively by using electronic medical records maintained by the Department of Nephrology at Osaka Medical and Pharmaceutical University Hospital. Cr, BUN, eGFR, urine protein-to-creatinine ratio, urinary NAG, urinary β2 MG, and patient characteristics (e.g., underlying diseases, age, sex, blood pressure, and body mass index) were searched from the electronic medical records and kidney biopsy database. No diagnosis of fatty liver was diagnosed by liver biopsy or ultrasonography.

### Kidney biopsy

Kidney specimens were obtained with a 16-gauge biopsy needle (Bard, New Providence, NJ). Specimens were fixed in 10% formalin, and the prepared sections were stained with hematoxylin–eosin, Masson trichrome, periodic acid silver methenamine, or periodic acid-Schiff. At least three pathologists evaluated each specimen.

### Definition of non-invasive fibrosis markers

The degree of liver fibrosis was evaluated by the FIB-4 index and NFS. FIB-4 was calculated using the following formula: age (years) × aspartate aminotransferase (AST, IU/L) / platelet (109/L) × √ alanine aminotransferase (ALT, IU/L). High FIB-4 index group was defined as 1.3 or higher^30^. NFS was calculated using the following formula: − 1.675 + 0.037 × age (years) + 0.094 × body mass index (kg/m2) + 1.13 × impaired fasting glucose/diabetes (yes = 1, no = 0) + 0.99 × AST (IU/L) / ALT (IU/L)—0.013 × platelet (109/L)—0.66 × albumin (g/dL).

### Calculation of eGFR

Levels of serum creatinine were measured in all samples using an enzymatic method in laboratories, and the values are represented using two decimal places. The eGFR of each patient was calculated using the following formula: 194 × serum creatinine − 1.094 × age − 0.287 × 0.739 (if female). The rate of change in eGFR was used to evaluate renal function in each period and was calculated using the following formula: ((eGFR 12 months after kidney biopsy—eGFR before kidney biopsy))/eGFR before kidney biopsy) × 100.

### Statistical analyses

Continuous variables are presented as medians. Comparisons were made between groups using the Mann–Whitney U test; categorical variables were presented as numbers (percentage) and compared using Pearson’s Chi-squared test and Fisher’s exact test, as appropriate. All analyses were performed using StatView (SAS Institute, Cary, CA, USA) and Excel software. Statistical significance was defined as *P* < 0.05.

## Data Availability

The data that support the findings of this study are available in the figshare at https://figshare.com/articles/dataset/raw_data_update_xlsx/19590235, but restrictions apply to the availability of these data, and so are not publicity available. Data are however available from the author upon reasonable request and with permission of figshare.
